# Stabilization of Bilateral Progressive Rheumatoid Corneal Melt with Infliximab

**DOI:** 10.1155/2012/173793

**Published:** 2012-05-30

**Authors:** Sheelah F. Antao, Tariq Ayoub, Hasan Tahir, Dipak N. Parmar

**Affiliations:** ^1^Department of Ophthalmology, Whipps Cross Hospital, London E11 1NR, UK; ^2^Academic Rheumatology and Osteoporosis Unit, Whipps Cross Hospital, Whipps Cross Road, Leytonstone, London E11 1NR, UK

## Abstract

*Purpose*. To report the use of infliximab in the rapid stabilization of a case of progressive, bilateral rheumatoid peripheral ulcerative keratitis (PUK) that failed to respond to conventional immunosuppressive therapy. *Methods*. A single interventional case report. *Results*. A patient with rheumatoid arthritis presented with bilateral PUK following a 2-month history of ocular discomfort and redness. His systemic prednisolone (PDN) and methotrexate (MTX) were increased and, despite an initial favorable response, bilateral recurrent corneal perforations ensued. Both eyes underwent cyanoacrylate glue repair, amniotic membrane transplantation (AMT), and penetrating keratoplasty (PKP). Recurrence of the disease and bilateral perforations of the second PKP in both eyes prompted administration of intravenous infliximab immediately after the fourth PKP. The disease activity rapidly settled in both eyes, and at eighteen-month followup, after 12 infliximab infusions, the PUK remains quiescent with no further graft thinning or perforation. *Conclusion*. Infliximab can be used to arrest the progression of severe bilateral rheumatoid PUK in cases that are refractory to conventional treatment.

## 1. Introduction

Peripheral ulcerative keratitis (PUK) is an uncommon inflammatory condition that is associated with collagen vascular diseases such as rheumatoid arthritis (RA) [1]. Conventional systemic immunosuppressive therapy may fail and lead to relentlessly progressive disease with corneal perforation and blindness. Intravenous infliximab has been reported to halt the progression of PUK in RA and in other destructive inflammatory corneal conditions [2] including Mooren's ulcer [3]. We describe the use of infliximab to stabilize progressive and recurrent corneal melting in bilateral tectonic penetrating keratoplasties for PUK associated with RA. To the best of our knowledge, this would not appear to have been reported before.

## 2. Materials and Methods

### 2.1. Case Report

A 47-year-old Sri Lankan male presented in October 2006 with a 3-month history of bilateral red watery eyes with a foreign body sensation. He had RA, which was quiescent at presentation for which he was taking oral diclofenac, prednisolone (PDN), and methotrexate (MTX) with calcium and folic acid supplements.

On examination, his best corrected visual acuity (BCVA) was 6/19 right eye (OD) and 6/9 left eye (OS). There was an inferonasal peripheral crescent-shaped area of corneal thinning (80%) forming a gutter from 3 to 6 o'clock, 1.4 mm wide in the right eye, while in the left eye there was a smaller inferonasal gutter with 20% thinning. His PDN and MTX were increased and hourly topical carmellose sodium 0.5% added. Initial reduction of ocular discomfort prompted reduction of his systemic medication, but on review within 9 days BCVA OD was reduced to 6/38 and the right anterior chamber was shallow with a small corneal perforation noted in the corneal gutter. This was plugged with iris and not actively leaking. The perforation was repaired with corneal cyanoacrylate glue and a bandage contact lens (BCL) was placed for comfort and exchanged at 6 weekly intervals thereafter. Slow tapering of his systemic medication was attempted again. On review 4 months later in February 2007, a small leak from the previous perforation was noted. After further cyanoacetate glue repair, he underwent multilayered amniotic membrane graft (AMG) to the right eye in March 2007.

**Figure 1:**
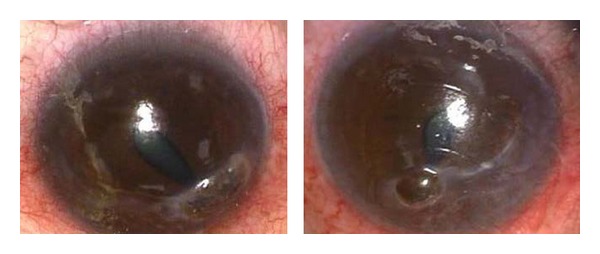
Bilateral inferonasal crescent-shaped corneal thinning with perforation and iris prolapse, June 2009.

**Figure 2:**
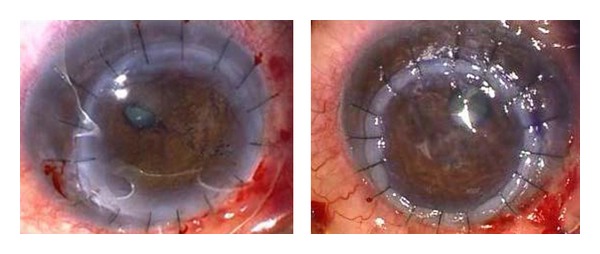
Bilateral iris reconstruction and inferonasal tectonic keratoplasties, June 2009.

Initially his postoperative course was satisfactory but attendance at follow-up appointments was poor. In June 2009, he attended with marked discomfort and redness of both eyes and BCVA of 6/15 OD and 6/7 OS. He was systemically well with no signs of active RA. He was found to have marked bilateral inferior corneal stromal thinning with perforations in the inferotemporal mid-periphery and a small iris prolapse plugging the hole in both eyes (Figure 1). Bilateral emergency tectonic penetrating keratoplasties (PKP) were performed, decentered infero-nasally in order to replace the thinned cornea (Figure 2). Lamellar grafts were initially attempted but was converted to a PKP as a satisfactory lamellar plane was impossible to create due to a soft eye from the perforations. His systemic medications were again increased.

One week postoperatively, BCVA was 6/38 OD and 6/19 OS but one month later he presented with recurrent, bilateral paracentral corneal melts. Glue repair was again performed and topical cyclosporine 0.5% twice daily was added to both eyes. He subsequently underwent a further triple-layered AMG and second PKP to both eyes in September 2009.

**Figure 3:**
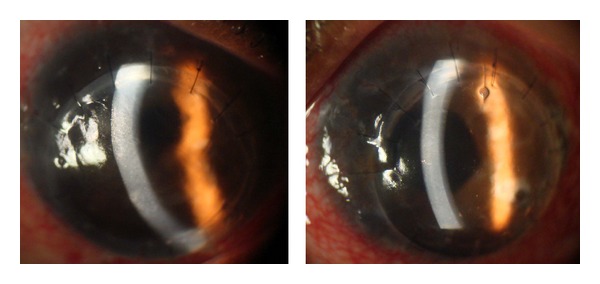
Right and left eyes after repeated penetrating keratoplasty and cataract extraction with intraocular lens implant, September 2010.

Infliximab (5 mg/kg intravenously) was commenced on the first postoperative day and he continued to receive cycles at the same dose at weeks 2 and 6 and then every 8 weeks, while PDN and MTX were gradually reduced. Since he was from an area of high prevalence of tuberculosis, prophylactic isoniazid was commenced. He underwent uncomplicated phacoemulsification with posterior chamber intraocular implantation in both eyes in June 2010. Eighteen months following infliximab therapy both eyes remained quiet with no signs of further corneal melting. The BCVA was 6/48 in both eyes, limited by subepithelial haze and thinning of his corneal grafts (Figure 3).

## 3. Discussion

Peripheral ulcerative keratitis is a rare corneal disease associated with rheumatoid arthritis and is indicative of active local vasculitis [1]. Deregulated proteolytic mechanisms within the corneal stroma cause rapid corneal thinning and perforation, which can be aggressive and recurrent. The biochemical pathways involved are numerous and not yet fully understood.

Healthy corneal tissue contains proteinases, which are upregulated and activated in the damaged cornea, their synthesis being increased by proinflammatory cytokines such as tumour necrosis factor-alpha (TNF*α*) and interleukin-1 (IL-1) [4]. These proteinases are regulated by specific inhibitors in healthy ocular surface tissues and tear film, thus preventing excessive degradation of healthy stroma. Unregulated matrix metalloproteinases (MMPs) are generated through several inflammatory pathways and considered to be largely responsible for the destruction of the extracellular matrix. The increased expression and activity of a wide range of MMPs, particularly MMP-9, a gelatinase which degrades collagen [5], has been demonstrated in melted human corneal samples [4] and in veterinary studies.

Deficiencies in the corneal surface environment, including keratoconjunctivitis sicca, may initiate stromal lysis, thus increasing the risk of recurrent melts and graft failure [5]. In humans with dry eye symptoms, MMP-9, IL-1*β*, and TNF-*α* expression and activity in conjunctival epithelial cells are higher than in controls and correlate well with clinical parameters [6]. Topical cyclosporine A has been established as an effective treatment for dry eye and was thus used in our patient to dampen the local inflammatory response.

TNF*α* is a proinflammatory cytokine that is a major mediator of inflammation, immunity, and apoptosis. It increases vascular endothelial permeability, enhances leucocyte chemotaxis from blood vessels into tissues, and increases expression of cellular adhesion molecules. In human corneal epithelial cell cultures, TNF*α* stimulates MMP-9 activity in a dose-dependent manner [4]. This is key evidence that TNF*α* may contribute to the keratolytic pathway and could therefore be an important therapeutic target in treating corneal melts.

The keratolytic processes that cause corneal melting may persist even after PKP and are a common cause of graft failure. Graft survival is significantly improved with systemic immunosuppression but corneal melts may still recur despite the use of different agents. Individual variations in the immune response are likely to play an important role in clinical outcome.

The anti-TNF*α* agent infliximab is a chimeric monoclonal antibody, which prevents free- and membrane-bound TNF*α* binding to its cellular receptor and induces apoptosis in activated T-cells expressing TNF*α* [7]. It reduces upregulation of TNF*α* at sites of inflammation. Administration is by 8 weekly intravenous infusion after loading doses at 0, 2, and 6 weeks and in the UK requires supervision by a rheumatologist. It is currently used systemically to treat RA, ankylosing spondylitis, Crohn's disease, and psoriasis. We elected to use a higher dose (5 mg/kg) of infliximab rather than the standard (3 mg/kg) dose typically used for systemic rheumatoid arthritis in view of the bilateral involvement and advanced severity of corneal disease.

Another anti-TNF*α* agent, etanercept, has been reported to treat sterile corneal ulceration in inflammatory disease but current data is insufficient to establish which agent offers the greatest and most consistent therapeutic benefit. Infliximab has been increasingly used to treat refractory uveitis and at least one report has suggested favourability over etanercept [8]. In-depth profiling of the targets and pathways of action of these agents would help to determine their future roles in treating localized manifestations of systemic inflammatory disease.

This case demonstrates that infliximab can effectively stabilize active corneal rheumatoid melts resistant to conventional therapy. It is unique in successfully arresting bilateral progressive disease after multiple bilateral tectonic keratoplasties.
